# The histone variant *Sl*_H2A.Z regulates carotenoid biosynthesis and gene expression during tomato fruit ripening

**DOI:** 10.1038/s41438-021-00520-3

**Published:** 2021-04-01

**Authors:** Xuedong Yang, Xuelian Zhang, Youxin Yang, Hui Zhang, Weimin Zhu, Wen-Feng Nie

**Affiliations:** 1 Shanghai Key Laboratory of Protected Horticultural Technology, Horticulture Research Institute, Shanghai Academy of Agricultural Sciences, 201403 Shanghai, China; 2 Department of Horticulture, College of Agronomy, Jiangxi Agricultural University, 330045 Nanchang, Jiangxi China; 3 Department of Horticulture, College of Horticulture and Plant Protection, Yangzhou University, 225009 Yangzhou, Jiangsu China

**Keywords:** Genetics, Plant sciences

## Abstract

The conserved histone variant H2A.Z is essential for transcriptional regulation; defense responses; and various biological processes in plants, such as growth, development, and flowering. However, little is known about how H2A.Z affects the developmental process and ripening of tomato fruits. Here, we utilized the CRISPR/Cas9 gene-editing system to generate a *sl_hta9 sl_hta11* double-mutant, designated *sl_h2a.z*, and found that these two mutations led to a significant reduction in the fresh weight of tomato fruits. Subsequent messenger RNA (mRNA)-seq results showed that dysfunction of *Sl*_H2A.Z has profound effects on the reprogramming of genome-wide gene expression at different developmental stages of tomato fruits, indicating a ripening-dependent correlation between *Sl*_H2A.Z and gene expression regulation in tomato fruits. In addition, the expression of three genes, *SlPSY1*, *SlPDS*, and *SlVDE*, encoding the key enzymes in the biosynthesis pathway of carotenoids, was significantly upregulated in the later ripening stages, which was consistent with the increased contents of carotenoids in *sl_h2a.z* double-mutant fruits. Overall, our study reveals a role of *Sl*_H2A.Z in the regulation of carotenoids and provides a resource for the study of *Sl*_H2A.Z-dependent gene expression regulation. Hence, our results provide a link between epigenetic regulation via histone variants and fruit development, suggesting a conceptual framework to understand how histone variants regulate tomato fruit quality.

## Introduction

In eukaryotes, the packaging of genomic DNA into chromatin is the main form of regulation of gene transcription^1–3^. As the basic unit of chromatin, a nucleosome is composed of an octamer of histones H2A, H2B, H3, and H4, with 146 base pairs of DNA wrapped around the octamer^4^. Chromatin is usually defined as transcriptionally active euchromatin and compacted inactive heterochromatin, both of which are mainly established and maintained by histone modifications and histone variants^5,6^. Eukaryotic genomes contain several histone variants that are primarily found among the members of the histone H3 and H2A families^7^. Among these variants, histone variant H2A.Z in the H2A family has been thoroughly studied in several organisms^8–12^. In the genome, H2A.Z is greatly enriched at the transcription start site (TSS) of genes^5,10,13^, which is important for maintaining the activity of genes, reflecting a significant role in the regulation of transcription^3,7,14^.

Histone variant H2A.Z has been proposed to have either a promotive or repressive effect on transcription, depending on its localization within genes^15^. In *Arabidopsis*, H2A.Z within gene bodies has a strong repressive effect on the transcription of stress-response genes^15^. H2A.Z within the gene body represses the expression of stress-response genes under noninductive conditions, while H2A.Z deposited at the TSS positively or negatively regulates transcription in rice^16^. H2A.Z occupancy, rather than its distribution, changes simultaneously with transcriptional changes and regulates active DNA demethylation^6^. Moreover, H2A.Z influences nucleosome stability and chromatin structure to modulate gene expression^17^. The genome-wide role of H2A.Z in chromatin structure and gene regulation is well established. H2A.Z also has defined regulatory roles in the expression of specific genes involved in development and adaptation^18^, which, along with its incorporation into chromatin, affirm that H2A.Z is central to transcriptional regulation during developmental and environmental responses^19^.

In *Arabidopsis*, there are three functional genes encoding H2A.Z: HISTONE H2A8 (*AtHTA8*), *AtHTA9*, and *AtHTA11*^20^. In rice, there are three genes (*HTA705*, *HTA712*, and *HTA713*) encoding H2A.Z^12^. *Arabidopsis hta9-1 hta11-1* double mutants exhibit early flowering and have serrated leaves, and such mutations induce the expression of thousands of genes^3^; however, the *Arabidopsis hta9 hta11* double mutant exhibits a less severe phenotype due to the redundancy of HTA8, and this phenotype is confirmed in nearly null *h2a.z* triple mutants^21^. More recently, an *Arabidopsis* complete *h2a.z-2* knockout triple mutant generated by CRISPR/Cas9 displays severe growth defects^6^, indicating the importance of H2A.Z in the regulating plant growth and development. Notably, because *Arabidopsis*, a model plant species, lacks multiple horticultural processes and traits, such as the production and ripening of fresh fruits, the roles of H2A.Z in regulating fresh fruits of horticultural crop species remain unknown.

Tomato (*Solanum lycopersicum*) fruits, which are rich in nutrients and have unique flavor, is a widely used model species for studying the ripening and quality of fleshy fruits. As seed-bearing structures of flowering plants, edible fruits are indispensable for humans. Fruits provide important nutrients, including vitamins, carbohydrates, dietary fiber and multiple active substances, such as carotenoids and flavonoids. Moreover, fruit ripening is the key step for the improvement of fruit quality, which involves various physiological, biochemical and structural changes, accompanied by dramatic alterations in color, pigment contents, and carbohydrate contents^22,23^.

Various studies have suggested that fruit ripening can be driven by multiple regulators at the epigenomic and transcriptomic levels. It has been shown that fruit ripening is influenced by DNA methylation patterns and levels^24,25^ and that DNA methylation affects the expression of ripening-related genes^26,27^, suggesting that the activity of numerous metabolic pathways during ripening is potentially associated with DNA methylation and its subsequent effects on the expression of corresponding genes^28^. Similar to the role of DNA methylation in regulating fruit ripening, histone acetylation was recently reported to be involved in regulating fruit ripening. Silencing the histone deacetylase-encoding gene *SlHDT3* inhibits fruit ripening by suppressing ethylene biosynthesis and carotenoid accumulation^29^. Histone acetylation and H2A.Z deposition are correlated and occur at many genomic regions in *Arabidopsis*^6^. The chromatin-remodeling complex SWR1 is recruited to chromatin by recognizing histone acetylation marks and then depositing H2A.Z to the targeted regions to activate DNA demethylation^6^. DNA methylation, histone acetylation and H2A.Z are related factors in the epigenome. However, the roles of histone variant H2A.Z in the development and ripening of tomato fruit are still unknown.

Here, we characterize the histone variant *Sl*_H2A.Z and elucidate the roles of *Sl*_H2A.Z in the development and ripening of tomato fruits. We found that the tomato genome contains three putative *Sl*_H2A.Z-encoding genes, referred to as *Sl_HTA8*, *Sl_HTA9*, and *Sl_HTA11*. To investigate the genome-wide effect of *Sl*_H2A.Z on gene expression during fruit ripening, we performed mRNA-seq of tomato fruits of both WT and *sl_h2a.z* mutant plants at four different ripening stages. We found that double mutations of *Sl*_H2A.Z have great effects on the reprogramming of genome-wide gene expression in tomato fruits and that *Sl*_H2A.Z mutations reduce the fresh weight of tomato fruits and increase the contents of carotenoids. Hence, our study provides a valuable resource for the investigation of genes whose expression is regulated by the histone variant *Sl*_H2A.Z in horticultural crop species.

## Results

### Characterization of H2A proteins in *S. lycopersicum*

**Fig. 1:**
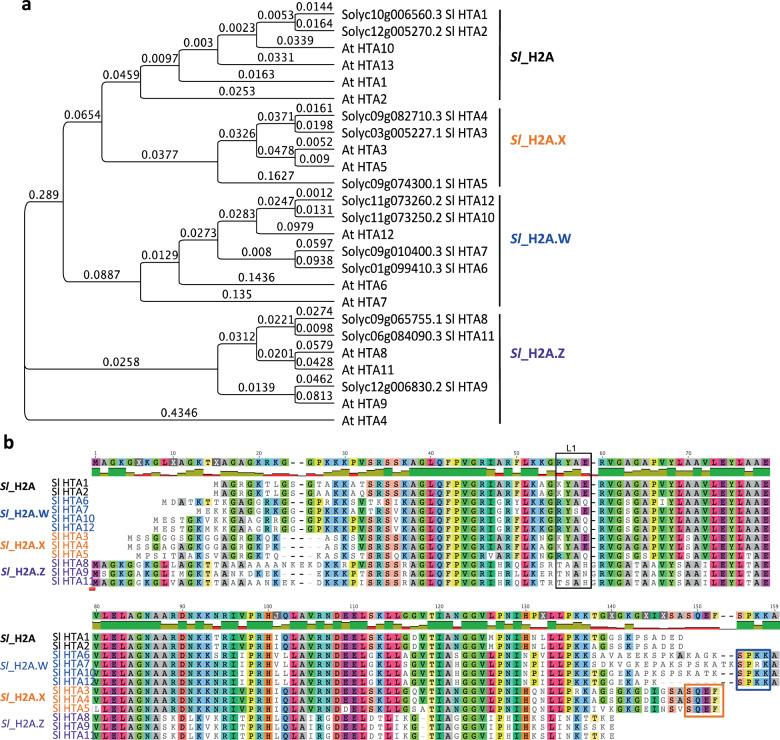
Characterization of tomato H2A family proteins. **a** Phylogenetic tree of H2A family proteins from *Arabidopsis thaliana (At)* and *S. lycopersicum (Sl)*. The phylogenetic tree is generated by Geneious software, according to genetic distance model (Jukes-Cantor). The number of the branch in the phylogenetic tree indicates the chance of amino acid substitution per site. **b** Alignment of *S. lycopersicum (Sl)* histone H2A variants. *Sl*_H2A variants are divided into four classes based on their conserved motifs in their C-terminal tails. *Sl*_H2A.X and *Sl*_H2A.W have the specific motifs SQEF and SPKK, respectively. *Sl*_H2A.Z variants have the shortest C-terminal tails among *Sl*_H2A variants. The L1 loop of *Sl*_H2A.Z is different from the other H2A variants^7^.

There are four H2A variants, named H2A, H2A.X, H2A.W and H2A.Z, and these variants define different genomic features, contributing to the overall genomic organization of *Arabidopsis*^5,30^. In this study, we found that the *S. lycopersicum* genome included 12 genes encoding histone *Sl*_H2A variants, which were grouped into four classes according to their C-terminal conserved motif: *Sl*_H2A and *Sl*_H2A.X, *Sl*_H2A.W, and *Sl*_H2A.Z (Fig. 1a, b). Two *Sl_HTA* genes, *Sl_HTA1* (Solyc10g006560) and *Sl_HTA2* (Solyc12g005270), encoded SlH2A, and three *Sl_HTA* genes, *Sl_HTA3* (Solyc03g005227), *Sl_HTA4* (Solyc09g082710), and *Sl_HTA5* (Solyc09g074300), were *Sl*_H2A.X-encoding genes, which harbor the specific SQEF motif^5^. Furthermore, *Sl_HTA6* (Solyc01g099410), *Sl_HTA7* (Solyc09g010400), *Sl_HTA10* (Solyc11g073250), and *Sl_HTA12* (Solyc11g073260) were identified as encoding *Sl*_H2A.W (Fig. 1a, b), according to the presence of the SPKK motif in the C-terminal region in *Arabidopsis*^31,32^. Three of the twelve *Sl_HTA* genes, *Sl_HTA8* (Solyc09g065755), *Sl_HTA9* (Solyc12g006830), and *Sl_HTA11* (Solyc06g084090), were classified as *Sl*_H2A.Z-encoding genes (Fig. 1a), with the shortest C-terminal regions among the H2A variants and different L1-loops^7^ (Fig. 1b).

### Generation of *sl_hta9 sl_hta11* double-mutant by the CRISPR/Cas9 gene-editing system

**Fig. 2:**
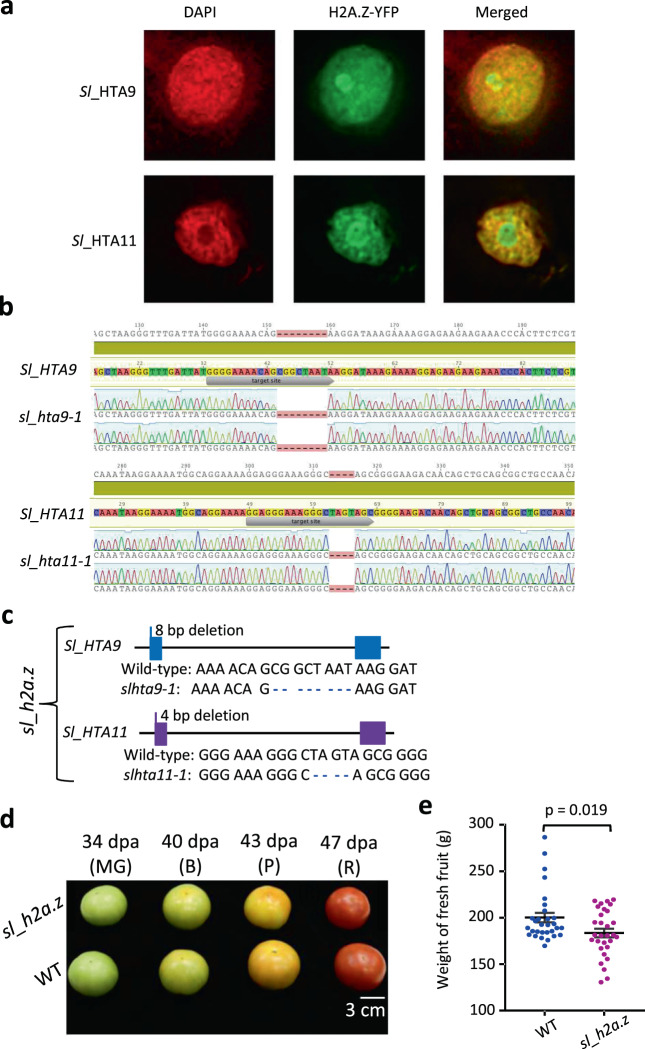
The localization of *Sl*_H2A.Z and generation of *sl_h2a.z* double-mutant. **a** The nucleus localization of *Sl*_HTA9 and *Sl*_HTA11 in *N. benthamiana* epidermal cells. **b** Sequencing results showing mutation in *sl_hta9-1* or *sl_hta11-1* single mutant generated by CRISPR/Cas9. The genotype in the T2 seedlings of *sl_hta9-1* or *sl_hta11-1* mutant was confirmed by sequencing. **c** Schematic diagrams showing the position and nature of mutations in the *sl_h2a.z* double-mutant. bp, base pair. The *sl_h2a.z* double-mutant was generated by crossing the *sl_hta9-1* single mutant with *sl_hta11-1* single mutant. The homozygous *sl_h2a.z* double-mutant was genotyped by sequencing. **d** Image of fruits in WT and *sl_h2a.z* at 34 dpa (MG stage), 40 dpa (B stage), 43 dpa (P stage), and 47 dpa (R stage). dpa, days after pollination. MG, mature green. B, breaker. P, pink. R, red. **e** The average weight of fresh fruits in WT and *sl_h2a.z*. *P = 0.019 (*n* = 30), compared with WT control fruits (2-tailed *t*-test).

In *Arabidopsis*, the *hta9-1 hta11-1* double-mutant flowers early and has serrated leaves, and the expression of more than two thousand genes is misregulated^3^. We first found that both *Sl*_HTA9 and *Sl*_HTA11 were located in the nucleus (Fig. 2a). To detect whether *Sl*_H2A.Z regulates tomato fruit ripening, using the CRISPR/Cas9 gene-editing system, we generated a stable loss-of-function *sl_hta9 sl_hta11* double-mutant, referred to as *sl_h2a.z* (Fig. 2b, c)^33^. We found 8- and 4-bp deletion mutations in *sl_hta9-1* and *sl_hta11-1*, respectively (Fig. 2b, c). We did not separate the homozygous line of the *sl_hat9 sl_hta11 sl_hta8* triple mutant, which was probably due to the lethality of the triple mutant, since severe defects in growth and development have already been shown in the complete *h2a.z* knockout triple mutant in *Arabidopsis*^6^.

### Double mutations of *Sl_H2A.Z* decreased the fresh weight of tomato fruits

We compared the tomato fruits of the WT and *sl_h2a.z* double-mutant plants at four stages of tomato fruit ripening: at 34 days after pollination (dpa), referred to as mature green (MG) stage; at 40 dpa, referred to as the breaker (B) stage; at 43 dpa, referred to as the pink (P) stage; and at 47 dpa, referred to as the red (R) stage (Fig. 2d). The dysfunction of *Sl*_H2A.Z did not visibly change the color of the tomato fruits compared with the WT tomato fruits (Fig. 2d). However, the average weight of fresh fruits significantly decreased in the *sl_h2a.z* double-mutant fruits compared with the WT fruits (Fig. 2e), suggesting that histone variant H2A.Z regulates the development of tomato fruits. The biomass of 4-week-old seedlings was reduced in the *sl_h2a.z* double-mutant plants (Fig. S1), which is consistent with the role of H2A.Z in controlling the growth and development of *Arabidopsis* leaves^21,34^.

### Dysfunction of *Sl_H2A.Z* and ripening influence the transcript levels of genes in tomato fruits

**Fig. 3:**
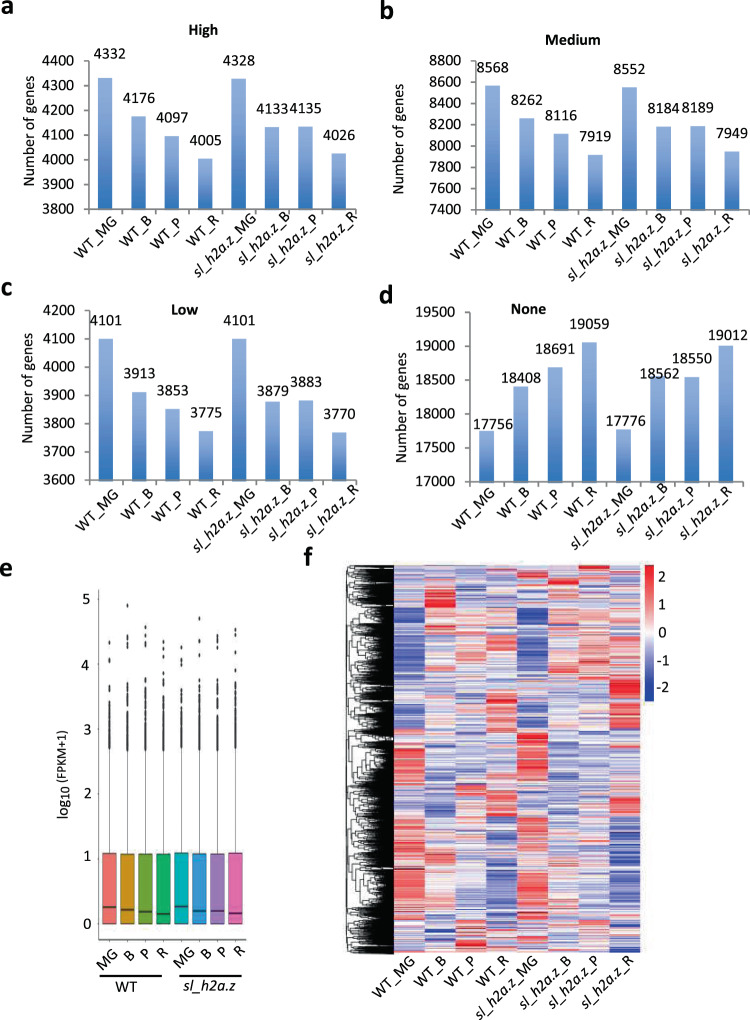
Fruit ripening reduces the genome-wide transcript level. **a**–**d** Numbers of genes in the indicated samples at different percentiles of expression level, referred to as 25% high expression (**a**), medium expression (**b**), low expression (**c**), and none expression (**d**). **e** Average of expression level of all the annotated genes in *S. lycopersicum* genome in WT and *sl_h2a.z* fruits at the four ripening stages. **f** Heatmap representation of expression level of all the annotated genes in *S. lycopersicum* genome.

Incorporation of the histone variant H2A.Z into nucleosomes by the SWR1 chromatin-remodeling complex is a critical step in eukaryotic gene regulation^3,15^. To elucidate how gene expression is regulated by *Sl*_H2A.Z during fruit ripening, we carried out a global transcriptome analysis of the tomato fruits at four different fruit ripening stages in both WT and *sl_h2a.z* plants by mRNA-seq (Fig. 2d). The sequencing quality for all the samples was quite high the raw sequencing data were discarded (Supplemental Tables 1 and 2). The principal component analysis (PCA) data indicated a high consistency among the three biological replicates in each group (Fig. S2a). The Pearson correlation (*R*^2^) values between replicates exceeded 95%, indicating high consistency among biological replicates (Fig. S2b). Genes with fragments per kilobase of transcript per million mapped reads (FPKM) values of <1 were defined as barely expressed genes^14,35^. We divided the expressed genes (FPKM ≥ 1) into three subgroups according to the FPKM percentiles: low expression level (<25th percentile), medium expression level (between the 25th and 75th percentiles), and high expression level (>75th percentile). Interestingly, we found that the numbers of expressed genes (FPKM ≥ 1) gradually decreased during tomato fruit ripening from the MG to R stages, while the numbers of rarely expressed genes gradually increased (Fig. 3a–d). The results presented in the boxplot also confirmed that the transcript levels of genes were decreased (Fig. 3e), suggesting that multiple genes became less inactive with fruit ripening. Double mutations of *Sl*_H2A.Z did not largely influence the transcript levels of genes in the tomato fruits at any developmental stage, with the exception of the transition from the B to P stage: the trend by which the numbers of expressed genes in the tomato fruits decreased from stage B to stage P was abolished in the *sl_h2a.z* double-mutant fruits when compared with the WT control fruits (Fig. 3a–d). The heatmap representation of the transcript level showed that both dysfunction of *Sl_H2A.Z* and ripening obviously reprogrammed genome-wide gene expression patterns (Fig. 3f).

### Dysfunction of *Sl*_H2A.Z induced numerous differentially expressed genes during tomato fruit ripening

**Fig. 4:**
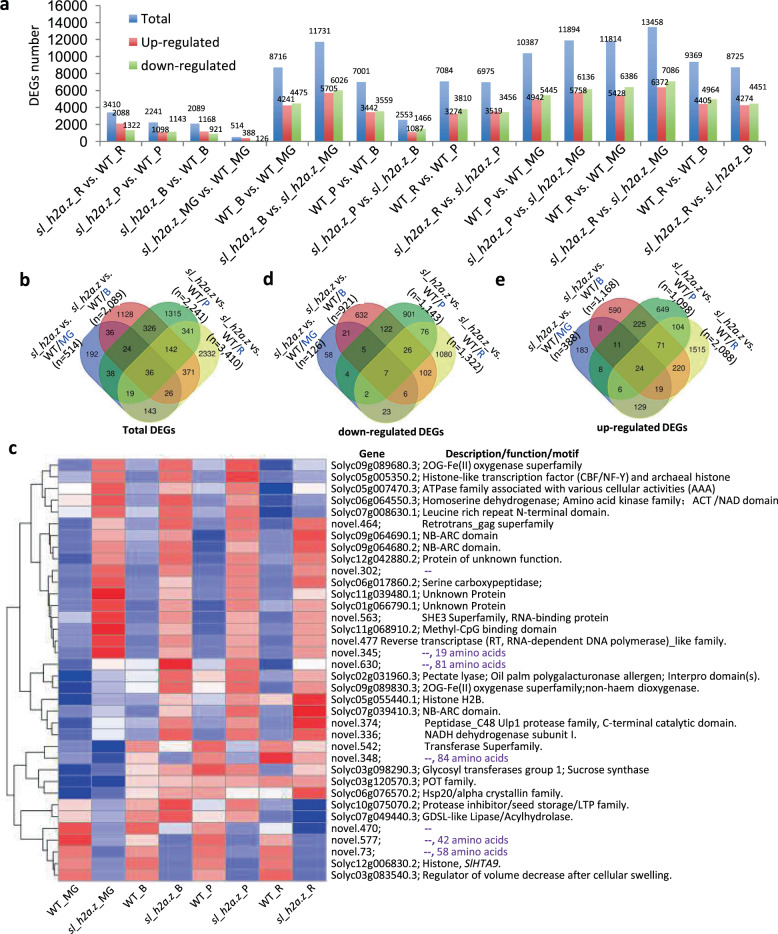
Characterization of DEGs identified in the tomato fruits of *sl_h2a.z* double-mutant at the MG, B, P, and R stages. **a** Numbers of DEGs identified in the comparisons between the indicated samples shown in Fig. 2c. **b**, **d**, **e** Overlap of total DEGs (**b**), overlap of downregulated DEGs (**d**), and overlap of upregulated DEGs (**e**) at four ripening stages in *sl_h2a.z* double-mutant fruits when compared with WT fruits. **c** Heatmap representation of expression levels in the commonly shared DEGs (shown in **b**) at all the four ripening stages in WT and *sl_h2a.z* fruits.

Overall, our analysis showed a massive transcriptional reprogramming in response to *Sl_H2A.Z* mutations (Fig. 3f). We found that dysfunction of *Sl*_H2A.Z resulted in dysregulation of the expression of multiple genes and novel transcripts in tomato fruits during ripening (Tables S3 and S4). Unlike in WT tomato fruits, the mutations in *sl_h2a.z* in the fruits of the mutant resulted in 388, 1168, 1098, and 2088 differentially expressed genes (DEGs) whose expression was upregulated at the MG, B, P, and R stages, respectively (Fig. 4a). There were 126, 921, 1143, and 1322 DEGs whose expression was downregulated in the *sl_h2a.z* tomato fruits at the MG, B, P, and R stages, respectively (Fig. 4a). These results suggest that the expression of numerous genes at different developmental stages of tomato fruits was *Sl*_H2A.Z-dependent. Moreover, there were 11,814 and 13,458 DEGs in the WT and *sl_h2a.z* fruits at the R stage compared with the MG stage, respectively (Fig. 4a), and there were 9369 and 8725 DEGs in the WT and *sl_h2a.z* fruits at the R stage compared with the B stage, respectively (Fig. 4a). There were 7084 and 6975 DEGs in the WT and *sl_h2a.z* fruits at the R stage compared with the P stage, respectively (Fig. 4a). In the WT fruits, the number of DEGs between the R stage and MG stage was higher than the number between the R stage and B (or P) stage, indicating that gene expression was largely reprogrammed in the process of tomato fruit ripening. Notably, 2553 DEGs were identified in the comparison between the B stage and P stage in the *sl_h2a.z* fruits, which was much lower than that in the WT fruits (7001 DEGs) (Fig. 4a), suggesting *Sl*_H2A.Z is essential in the developmental transition of tomato fruits from the B stage to the P stage. In total, 514, 2089, 2241 and 3410 DEGs were identified because of the dysfunction of *Sl*_H2A.Z at the MG, B, P, and R fruit ripening stages, respectively, showing gradually increasing numbers of reprogrammed genes with increasing fruit ripening (Fig. 4a). Finally, 36 DEGs were common between the WT and *sl_h2a.z* fruits at the four fruit ripening stages (Fig. 4b, c and Tables S3 and S4), among which the expression of 7 genes was downregulated (Fig. 4d) and the expression of 24 genes was upregulated (Fig. 4e) at all four fruit ripening stages.

### *Sl*_H2A.Z reprogrammed the expression patterns of ripening-dependent genes

To understand the regulation of ripening and development in the *sl_h2a.z* double-mutant fruits, we examined ripening-dependent mRNA transcripts in the WT and *sl_h2a.z* mutant fruits at the four different ripening stages. There were 6386 and 7086 DEGs whose expression was downregulated in the WT and *sl_h2a.z* fruits, respectively, and the expression of 5428 and 6372 genes was upregulated in the WT and *sl_h2a.z* fruits, respectively, at the R stage compared to the MG stage (Fig. S3a, b). We calculated the expression levels of the DEGs identified whose expression was downregulated or upregulated in the WT_R vs. WT_MG comparison across all the samples. We found that the expression level of genes in both the WT and *sl_h2a.z* fruits showed a decreasing trend during fruit ripening, reflected by a cluster of DEGs whose expression was downregulated (Fig. S3c, d). Moreover, the expression level of genes in both the WT and *sl_h2a.z* fruits displayed an increasing trend during the fruit ripening process, reflected by a cluster of DEGs whose expression was upregulated (Fig. S3e, f). The dysfunction of *Sl*_H2A.Z largely caused misregulation of the expression patterns of genes in the mutant tomato fruits compared to the WT fruits at the corresponding ripening stage, as reflected by their heatmap representations (Fig. S3c, e) but not by the average transcript level represented in the boxplots (Fig. S3d, f).

### Characterizations of the DEGs regulated by ripening and H2A.Z in tomato fruits

To characterize the effects of fruit ripening on the expression of genes in both WT and *sl_h2a.z* fruits, we compared the DEGs identified from the comparisons among different ripening stages. In total, 2470 DEGs whose expression was upregulated were common among the WT tomato fruits at the B, P, and R stages compared to the MG stages (Fig. S4a, left panel), and 3834 DEGs whose expression was upregulated were common among the *sl_h2a.z* double-mutant tomato fruits at the B, P, and R stages compared to the MG stages (Fig. S4a, middle panel). In addition, 2,928 DEGs whose expression was downregulated were common in the WT tomato fruits at the B, P, and R stages compared to the MG stage (Fig. S4b, left panel), and 4141 DEGs whose expression was downregulated were common in the *sl_h2a.z* fruits at the B, P, and R stages compared to the MG stage (Fig. S4b, middle panel). The common DEGs whose expression was upregulated or downregulated in the B vs. MG, P vs. MG, and R vs. MG comparisons were referred to as ripening-dependent upregulated and downregulated DEGs, and the expression of the genes in these clusters was consistently induced by fruit ripening in both the WT and *sl_h2a.z* fruits.

Among the 2470 ripening-dependent DEGs in the WT fruits, 2243 DEGs overlapped with those in the *sl_h2a.z* fruits (Fig. S4a, right panel). Among the 2928 ripening-dependent DEGs whose expression was downregulated in the WT fruits, 2547 DEGs overlapped with those in the *sl_h2a.z* double mutant (Fig. S4b, right panel). There were 1,591 and 1594 DEGs whose expression was upregulated (Fig. S4a, right panel) and downregulated (Fig. S4b, right panel), respectively, showing specific dependence on the function of *Sl*_H2A.Z, suggesting that *Sl*_H2A.Z mutations may enable the expression of multiple genes during tomato fruit ripening.

To test whether the *Sl_H2A.Z* regulation of gene expression is dependent on different ripening stages, we analyzed the DEGs identified in the WT and *sl_h2a.z* fruits at different sequential developmental stages (i.e., B vs. MG, P vs. B, and R vs. P). From the MG stage to the B stage, the expression of 4241 and 5705 genes was upregulated in the WT and *sl_h2a.z* fruits, respectively (Fig. S5a, left panel), and the expression of 4475 and 6026 genes was downregulated, respectively (Fig. S5b, left panel). We found that 3732 DEGs whose expression was upregulated (approximately 87.8% of the number in the WT) and 3,933 DEGs whose expression was downregulated (~87.9% of the number in the WT) overlapped between the WT and *sl_h2a.z* tomato fruits (Fig. S5a, b, left panels). From the B stage to the P stage, the expression of 3442 and 1087 genes was upregulated in WT plants and *sl_h2a.z* fruits, respectively, and the expression of 3559 and 1466 genes was downregulated, respectively; 826 DEGs whose expression was upregulated (~24.0% of the number in the WT) and 1,169 DEGs whose expression was downregulated (~32.8% of the number in the WT) overlapped in the WT and *sl_h2a.z* tomato fruits (Fig. S5a, b, middle panels). From the P stage to the R stage, we found that the expression of 3274 and 3519 genes was upregulated in the WT and *sl_h2a.z* fruits, respectively,and that of 3810 and 3456 genes was downregulated, respectively; in addition, 1599 DEGs whose expression was upregulated (~48.8% of the number in the WT) and 1956 DEGs whose expression was downregulated (~51.3% of the number in the WT) overlapped between the WT and *sl_h2a.z* fruits (Fig. S5a, b, right panels).

### Determination of functionally enrichment in response to *Sl*_H2A.Z mutations and ripening

**Fig. 5:**
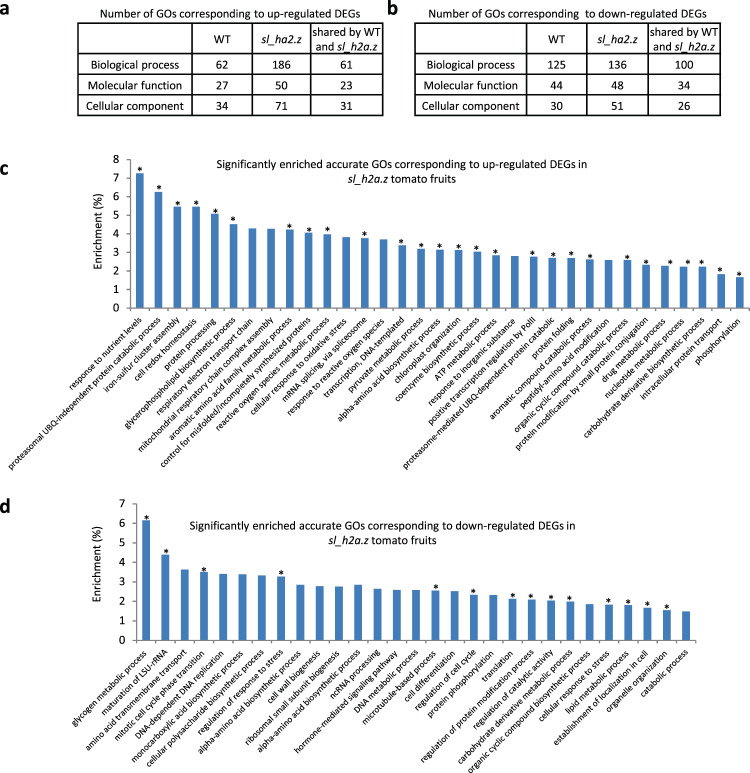
The enriched pathways identified in WT and *sl_h2a.z* double-mutant fruits during fruit ripening. **a** The numbers of the GOs identified in the ripening-dependent upregulated DEGs (identified in Supplemental Fig. 4a) in WT fruits and *sl_h2a.z* fruits. **b** The numbers of the GOs identified in the ripening-dependent downregulated DEGs (identified in Supplemental Fig. 4b) in WT fruits and *sl_h2a.z* fruits. **c** The top 33 enriched GOs corresponding to ripening-dependent upregulated DEGs in *sl_h2a.z* tomato fruits. **d** The topo 29 enriched GOs corresponding to ripening-dependent downregulated DEGs in *sl_h2a.z* tomato fruits. The asterisk indicates the GO specifically enriched in *sl_h2a.z* fruits, but not in the WT fruits in (**c**, **d**).

To investigate the function of DEGs induced by ripening and *Sl_H2A.Z* mutations, the DEGs were subjected to Gene Ontology (GO) functional enrichment classification. Generally, the GO classification results indicated that 62, 27, and 34 pathways corresponding to the biological process, molecular function, and cellular component categories, respectively (Fig. 5a and Table S5), were enriched in the ripening-dependent genes whose expression was upregulated in the WT fruits (Fig. S4a, left panel). We found that 186, 50, and 71 pathways corresponding to the biological process, molecular function, and cellular component categories, respectively (Fig. 5a and Table S5), were enriched in the ripening-dependent genes whose expression was upregulated in the *sl_h2a.z* fruits (Fig. S4a, middle panel). Accordingly, we found that 125, 44, and 30 GO terms were associated with pathways corresponding to biological processes, molecular functions, and cellular components, respectively (Fig. 5b), and were enriched in the ripening-dependent genes whose expression was downregulated in the WT fruits (Fig. S4b, left panel), and we found that 136, 48, and 51 pathways corresponding to biological processes, molecular functions, and cellular components, respectively (Fig. 5b), were enriched in ripening-dependent genes whose expression was downregulated in the *sl_h2a.z* fruits (Fig. S4b, middle panel). Additionally, we found that 61 GO terms in the biological process category, 23 GO terms in the molecular function category, and 31 GO terms in the cellular component category were enriched in both the WT- and *sl_h2a.z-*dependent DEGs whose expression was upregulated (Fig. 5a). For the GO terms corresponding to DEGs whose expression was downregulated, the WT and *sl_h2a.z* tomato fruits shared 100, 34, and 26 enriched pathways associated with the biological process, molecular function, and cellular component categories, respectively (Fig. 5b).

To identify the specific GO terms affected by the dysfunction of *Sl*_H2A.Z during tomato fruit ripening, the top 33 GO terms corresponding to ripening-dependent DEGs whose expression was upregulated and 29 GO terms corresponding to ripening-dependent DEGs whose expression was downregulated in the biological process category and that were enriched in *sl_h2a.z* tomato fruits are displayed in Fig. 5c, d. These enriched pathways were specifically associated with the categories “response to nutrient levels”, “proteasomal UBQ-independent protein catabolic process”, “iron-sulfur cluster assembly”, “cell redox homeostasis”, “reactive oxygen species metabolic process”, “mRNA splicing, via spliceosome”, “response to reactive oxygen species”, “positive transcription regulation by Pol II”, “organic cyclic compound catabolic process”, “protein modification by small protein conjugation”, “nucleotide metabolic process”, “carbohydrate derivative biosynthetic process”, “phosphorylation”, “maturation of LSU-rRNA”, “mitotic cell cycle phase transition”, “regulation of response to stress”, “microtubule-based process”, “regulation of cell cycle”, “translation” and so on (Fig. 5c, d), suggesting that dysfunction of *Sl*_H2A.Z influenced numerous biological processes involved in tomato fruit ripening.

### The total carotenoids increased in the *sl_h2a.z* tomato fruits

**Fig. 6:**
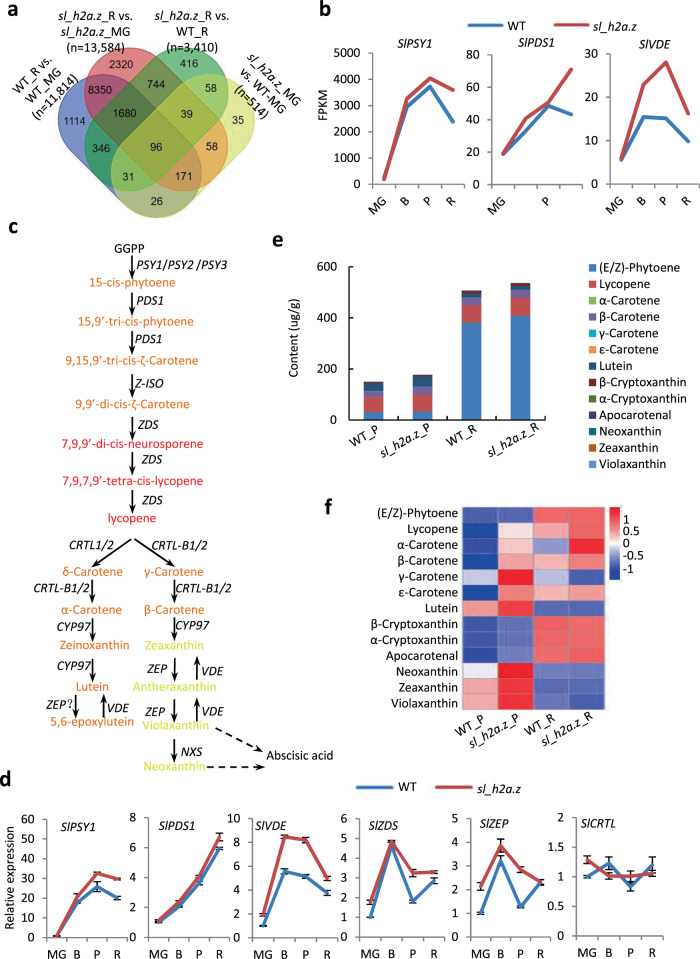
The *Sl_H2A.Z* mutations regulate the biosynthesis of carotenoids. **a** Overlapped DEGs among the indicated comparisons. The DEGs include the upregulated and downregulated DEGs. **b** The FPKM of the *SlPSY1*, *SlPDS1*, and *SlVDE* in the fruits in WT and *sl_h2a.z* backgrounds at the MG, B, P, and R stages. The values are the mean of the three biological replicates. **c** The pathway of carotenoids biosynthesis in plants. The key enzymes encoding genes in the pathway are marked. **d** qPCR detection of *SlPSY1*, *SlPDS*, *SlVDE*, *SlZDS*, *SlZEP*, and *SlCRTL* in the fruits of WT and *sl_h2a.z* at the MG, B, P, and R stages. Values are means ± SD of three biological replicates. **e** The contents of the indicated carotenoids in the fruits of WT and *sl_h2a.z* at the P and R stages. Values are means ± SD of four biological replicates. **f** Heatmap presentation of the contents of indicated carotenoids in the fruits of WT and *sl_h2a.z* at the P and R stages. The heatmap is generated by TBtool software based on the values of the contents of the indicated carotenoids.

Given that the expression of multiple genes and enriched GO pathways were largely induced by mutations in *Sl*_H2A.Z, whether *Sl*_H2A.Z regulates the quality of tomato fruits or not is still unknown. To identify the genes whose expression was simultaneously induced both by ripening and by mutations in *Sl*_H2A.Z, we identified the DEGs that overlapped between the MG stage and R stage in both the WT and *sl_h2a.z* fruits. Among the 10,297 DEGs common in the WT_R vs. WT_MG and in the *sl_h2a.z_*R vs. *sl_h2a.z*_MG comparisons, 1,680 were expressed only in the *sl_h2a.z_*R vs. WT_R comparison and not in the *sl_h2a.z_*MG vs. WT_MG comparison (Fig. 6a), suggesting that the expression of genes in this cluster was regulated simultaneously by ripening and *Sl*_H2A.Z. Among the 1680 DEGs, we found that the *PHYTOENE SYNTHASE 1* (*SlPSY1*) gene, encoding a key enzyme in the pathway of carotenoid biosynthesis, was abundantly expressed (FPKM > 2000 at the R stage) during the ripening process in both WT and *sl_h2a.z* fruits (Fig. 6b). The transcript level of *SlPSY1* largely increased in the *sl_h2a.z* fruits at the R stage, did the transcript levels of *PHYTOENE DESATURASE 1* (*SlPDS1*) and *VIOLAXANTHIN DE-EPOXIDASE* (*SlVDE*), compared to those in the WT fruits (Fig. 6b). *SlPDS1* and *SlVDE* are the genes encoding key enzymes involved in the carotenoid biosynthesis pathway. Using quantitative PCR (qPCR) assays, we confirmed the presence of several genes involved in the pathway of carotenoid biosynthesis, including *SlPSY1*, *SlPDS1*, *SlVDE*, ZETA-CAROTENE *DESATURASE* (*SlZDS*), and *ZEAXANTHIN EPOXIDASE* (*SlZEP*) (Fig. 6c), whose expression tended to increase in the *sl_h2a.z* mutant fruits (Fig. 6d). These results indicated that *Sl*_H2A.Z could regulate the biosynthesis of carotenoids in tomato fruits. We further measured the carotenoid contents in the WT and *sl_h2a.z* fruits at the P and R stages. The relative index (*R*^2^) from the equation of each tested component was >0.99, and the peak pattern of each standard of the indicated carotenoid was clear (Fig. S6a, b), indicating the relatively high accuracy and reliability of the detection method. We found that different carotenoids constituted different proportions in each tested sample (Fig. S6c). Lycopene, (E/Z)-phytoene, lutein, and β-carotene were the main components, and their contents were much higher than those of the other components in both the WT and *sl_h2a.z* fruits (Fig. 6e and Table S6). There are >600 carotenoids with natural structural variants, which are divided into lycopene, xanthophyll, and carotene compounds^36–38^. We found that the content of (E/Z)-phytoene dramatically increased at the R stage in both the WT and *sl_h2a.z* fruits compared to the fruits at the P stage, while dysfunction of *Sl*_H2A.Z did not obviously promote an increase in the (*E/Z*)-phytoene content in the fruits at either the P or R stage (Fig. 6e, f); the lycopene content slightly (but not significantly) increased in the *sl_h2a.z* fruits at both the P and R stages compared to those in the WT fruits (Fig. 6f and Table S6). The results showed that the contents of lutein, neoxanthin, violaxanthin, and neoxanthin decreased in the WT fruits at the R stage compared the P stage (Fig. 6f), suggesting that these kinds of carotenoids were degraded with increased ripening. The contents of α- and β-carotene increased in the *sl_h2a.z* fruits compared to the WT fruits at the P stage (Fig. 6f). The total content of all the tested carotenoids increased in the *sl_h2a.z* fruits compared to the WT fruits at both the P and R stages (Fig. 6e), which is consistent with the increased expression of genes encoding key enzymes in the carotenoid biosynthesis pathway (such as *SlPSY1*, *SlPDS1*, and *SlVDE*) in the *sl_h2a.z* mutant fruits.

## Discussion

### *Sl*_H2A.Z regulates the fresh weight of tomato fruits

H2A.Z has been widely known to play essential roles in multiple cellular processes by influencing the structure and environment of chromatin in eukaryotes^8,19,34,39^. In plants, H2A.Z regulates growth and development^6,21^, phase transitions^40^, and the response to temperature changes in the environment^18,41,42^. In this study, we first characterized members of the H2A family in tomato and identified three H2A.Z-encoding genes: *Sl_HTA8*, *Sl_HTA9* and *Sl_HTA11*. The results showed that mutations of two *Sl*_H2A.Z-encoding genes, *Sl_HTA9* and *Sl_HTA11*, influence the fresh weight of tomato fruits and the biomass of seedlings, which is consistent with the severe growth defects of *Arabidopsis* plants caused by the dysfunction of H2A.Z^6,21^. These results suggest that the conserved histone variant H2A.Z regulates not only vegetative growth (i.e., development of leaves) but also reproductive growth (i.e., development of fresh tomato fruits and flowering) of plants. Given that fruits and vegetables are important components of the human diet, our study on the functions of *Sl*_H2A.Z in regulating fresh weight and thereby increasing yield highlights the essential roles of histone variants and/or histone modifications in horticultural crop production^28^.

### Dysfunction of *Sl*_H2A.Z reprograms genome-wide gene expression

Tomato fruit ripening is a highly coordinated and genetically programmed process that includes initiation, maintenance and reorganization of multiple pathways at the transcriptional level to further drive ethylene signals and ripening-related events^26,43–46^. These various responses during fruit ripening are directly regulated by gene expression, which is strongly affected by chromatin structure. Chromatin organization can be altered through the incorporation of histone variant H2A.Z^5,8,11,47^. In this study, both the WT and *sl_h2a.z* fruits at four different ripening stages were collected for comparison. The mRNA-seq results suggest that *Sl*_H2A.Z may function in a wide range of communications during tomato fruit ripening and induce intricate genetic regulatory networks through its interaction with ripening. The DEGs identified in different comparisons based on genotype and at different fruit ripening stages, together with the enriched pathways revealed by GO analysis, reflect the key genes (i.e., those shown in Fig. 3) and coordinated regulatory patterns of different biological processes (i.e., those shown in Fig. 5) during the development and ripening of tomato fruits. These changes and variations between the WT and *sl_h2a.z* fruits reveal coordinated regulatory interactions between histone variants and the transcripts whose levels dynamically change during fruit ripening and provide valuable resources for future studies on fresh fruit biology driven by histone modifications (variants).

### *Sl*_H2A.Z shows dynamic regulation in tomato fruits

Thus far, we have presented comprehensive transcriptional results associated with ripening of fruits of both WT and *sl_h2a.z* mutant plants at four different ripening stages, revealing the spatial expression patterns associated with ripening in different genotypes at different ripening stages. Previous genomic studies on aging suggest that the expression of highly expressed and highly spliced genes is more likely to be downregulated with age in *Drosophila* photoreceptors^48^. Similar to how aging represses gene expression in *Drosophila*, the gradually increasing numbers of rarely expressed genes and the gradually decreasing numbers of expressed genes (Fig. 3a–d), as well as the reduced transcript levels during tomato fruit ripening (Fig. 3f), suggest that ripening not only reprograms whole-genome-wide gene expression patterns but also represses transcription. Finally, ripening may inactivate the expression of several subsets of genes that are not necessary for or negatively affect ripening. Moreover, similar trends were also found in the *sl_h2a.z* mutant fruits during ripening (Fig. 3). Unlike in the WT fruits, dysfunction of *Sl*_H2A.Z inhibits the changes in the gene expression patterns during ripening from the B stage to the P stage, suggesting that the function of *Sl*_H2A.Z have display time-dependent dynamic characteristics.

### Various functions of the histone variant H2A.Z in plants

In *Arabidopsis*, H2A.Z-containing nucleosomes provide thermosensory information that is used to coordinate the transcriptome under ambient temperature and accurately sense increasing environmental temperatures^18^. The transcriptome under warm ambient temperature is dependent upon members of the HSFA1 clade of *Arabidopsis* HSFs, which cause the rapid and dynamic removal of H2A.Z nucleosomes of target genes^42^. In *Brassica napus*, high temperatures inhibit the expression of the *FT* gene through dynamic regulation of H2A.Z and chromatin structure, thus resulting in delayed flowering^49^. Thermal stress effects on grain yield of *Brachypodium distachyon* occur via H2A.Z nucleosomes^41^. In the present study, we found that the numbers of DEGs identified in the *sl_h2a.z* mutant constitutively increased from 514 to 3,410 during ripening (from the MG stage to the R stage). These results indicate that interactions between H2A.Z and processes involving ripening may occur during the development of tomato fruits, and *Sl*_H2A.Z shows a developmental stage-dependent function in regulating gene expression, similar to how H2A.Z is sensitive to changing temperatures^18^. Various plant species show a rapid acceleration of flowering time in response to climate change, especially when experiencing extreme temperatures^50^. H2A.Z-containing nucleosomes provide specific characteristics applicable to wrapping of DNA, which indicates that there is a set of epigenetic mechanisms in plants to sense temperature changes through fluctuations in nucleosomes^18^. Chromatin remodeling and nucleosome assembly play an important role in sensing increases in ambient temperature^51,52^ to regulate flowering. Hence, these studies indicate that the functions of histone variant H2A.Z during the life cycle of plants, such as sensing changes in temperature, accelerating flowering time, and reprogramming gene expression, in tomato fruits are diverse.

### *Sl*_H2A.Z regulates carotenoid biosynthesis in tomato fruits

In this study, we report that dysfunction of *Sl*_H2A.Z increases the total content of carotenoids by regulating the expression of several genes involved in the carotenoid biosynthesis pathway. The deposition of lycopene is crucial for the characteristic red pigmentation of ripe tomato fruits, and β-carotene is associated with the change from green to red fruit color^53^. In our study, lycopene, lutein, and β-carotene, the three main carotenoids, increased in the *sl_h2a.z* fruits at the P stage. The increase in phytoene in both the WT and *sl_h2a.z* fruits was much higher than that in lycopene during ripening from the P stage to the R stage (Fig. 6e). This can be explained by the following factors: first, since lycopene is the product of the catalytic reaction of phytoene, it is possible that phytoene was not largely converted to lycopene at the sampling/testing stages; second, even though yellow phytoene is not as predominant as red lycopene, phytoene could still contribute to the pigmentation of tomato fruits. We propose that the increase in the contents of total carotenoids in the *sl_h2a.z* fruits is mainly induced by the upregulated expression of genes involved in the carotenoid biosynthesis pathway (i.e., *SlPSY1*). Unlike the content of the other three main carotenoids, the lutein content in the *sl_h2a.z* mutant fruit no longer increased at the R stage, which can be explained by the fact that the enzymes involved in the synthesis and catabolism of lutein are involved in multiple pathways (Fig. 6c). Fruits and vegetables are rich in antioxidant phytochemicals that prevent the risk of chronic diseases induced by oxidative stress^54–56^. Our results suggest that carotenoids function as antioxidants in tomato fruits and are regulated by the histone variant *Sl*_H2A.Z. Consistent with the role of carotenoids as antioxidants, the GO pathway “cellular response to oxidative stress” was specifically enriched in the *sl_h2a.z* fruits (Fig. 5c). Since flavor is an important trait of fruits, it is meaningful to investigate the factors influencing tomato flavor caused by dysfunction of histone variants and/or histone modifications in the future.

### Multiple factors associated with *Sl*_H2A.Z function during tomato fruit ripening

In addition to regulating the transcriptome, H2A.Z reportedly prevents DNA methylation at multiple loci^57^ and interacts with the DNA demethylase ROS1 to mediate DNA demethylation in *Arabidopsis*^6^. In tomato, decreased DNA methylation during fruit ripening is mainly induced by the upregulated expression of *SlDML2*^26,27^. Mutation of *SlDML2* inhibits the ripening of tomato fruits by preventing the DNA demethylation of ripening-related genes. Our results suggest that, unlike the function of SlDML2, the dysfunction of *Sl*_H2A.Z did not obviously influence tomato fruit ripening, which can be explained by the fact functional redundancy concerning *Sl*_HTA8 occurs and that H2A.Z directs only a subset of ROS1-targeted loci for DNA demethylation^6^. As detectors of methylation and demethylation, methyl-binding-domain-containing proteins are essential for the organization of methylation patterns in plants^58^. The expression of Solyc11g068910.2, encoding a methyl-CpG-binding-domain-containing protein, was downregulated in the *sl_h2a.z* double-mutant fruits at all four developmental and ripening stages (Fig. 4c), suggesting that MBD proteins may play a role in the histone variant-directed reprogramming of the transcriptome during tomato fruit ripening. The detailed function of MBD-containing proteins in fruit ripening needs to be further investigated in knockout mutants generated by the CRISPR/Cas9 gene-editing system.

In addition to the epigenetic and transcriptional regulators induced by H2A.Z, our results show that pathways related to cell redox and reactive oxygen species (ROS) are enriched in *Sl*_H2A.Z-dependent DEGs (Fig. 5c). Recent studies suggest that tomato fruit ripening is affected by regulation of redox signaling-mediated NOR transcription factors^59,60^. The redox reactions that function as regulators of activities of enzymes have been reported to be related to DNA methylation, histone methylation, histone acetylation, and chromatin remodeling^61^. ROS, important signaling factors induced in response to biotic and abiotic stresses in plants^62–64^, mainly control the cell redox state^65,66^. The response and metabolism of ROS in the enriched pathways identified in our study indicate that ROS may be involved in the regulation directed by the histone variant *Sl*_H2A.Z during the fruit ripening. The molecular mechanisms underlying ROS and the cell redox state resulting from the dysfunction of *Sl*_H2A.Z during fruit ripening may help us understand how the dynamic chromatin environment regulated by H2A.Z results in the production of various signals (i.e., secondary messengers) elicited in response to fruit development and ripening. These mechanisms will provide a key direction for breeding crop plants via epigenetic interference. Notably, our results indicate that protein modifications, including phosphorylation and ubiquitination^67^, are likely involved in the response to fruit ripening; these modifications are probably regulated by H2A.Z. The detailed functions and molecular mechanisms underlying protein modifications during fruit ripening are of interest and can potentially provide more strategies to improve the quality of fresh fruits.

## Experimental procedures

### Plant material and growth conditions

Wild-type (WT) seeds of the tomato (*S. lycopersicum*) inbred line 1479 were provided by the Shanghai Academy of Agricultural Sciences, Shanghai, China. All the tomato plants were grown in a greenhouse at the Shanghai Academy of Agricultural Sciences, Shanghai, China.

### Protein analysis

Sequences of *A. thaliana* histone proteins of the H2A family were obtained from the TAIR website (https://www.arabidopsis.org/)^5^. Sequences of *S. lycopersicum* histone proteins of the H2A family were obtained from the Sol Genomics Network website (https://solgenomics.net/). The histone H2A variants of *S. lycopersicum* and *A. thaliana* were aligned using Geneious software. Phylogenetic tree analysis of the histone H2As of *S. lycopersicum* and *A. thaliana* was also performed by Geneious software.

### Generation of *sl_h2a.z* double-mutant by CRISPR

Two 20-bp sgRNA oligos, 5ʹ-GGAGGGAAAGGGCTAGTAGC-3ʹ and 5ʹ- GGGGAAAACAGCGGCTAATA-3ʹ, targeted to *Sl_HTA11* (Solyc06g084090) or *Sl_HTA9* (Solyc12g006830), respectively, were cloned into the CRISPR/Cas9 gene-editing vector 18T-*SlU6*-chim. The cassette, which included chimeric RNA driven by the *SlU6* promoter and Cas9 driven by the *35S* promoter, was subcloned into a pCAMBIA1300 binary vector^33^. The constructs were subsequently transformed into tomato inbred line 1479 via *Agrobacterium* infection of leaf explants. Seeds from T2 plants contained homozygous *sl_hta9* or *sl_hta11* mutation were genotyped by sequencing. The construct in the T1 seedlings of the *sl_hta9-1* or *sl_hta11-1* mutant was isolated via selfing, which was confirmed by PCR. Finally, we generated *sl_h2a.z* double-mutant by crossing the *sl_hta9-1* and *sl_hta11-1* mutants and genotyped the *sl_h2a.z* double-mutant by sequencing to obtain the homozygous *sl_h2a.z* double-mutant.

### Detection of fresh fruit weight and measurements of aboveground biomass of seedlings

To measure the fresh weight of tomato fruits of the WT and *sl_h2a.z* plants, 15 plants of each genotype were selected. At 47 dpa, two fruits from the second panicle per plant were harvested. The average weight of the fresh fruits was then calculated (*n* = 30). To measure the biomass of the WT and *sl_h2a.z* plants, aboveground parts of 4-week-old seedlings were harvested and measured. Three biological replicates were included. For each biological replicate, the average weight of the aboveground portion of 12 seedlings was calculated.

### Nuclear localization assays

*Sl*_*HTA11* and *Sl*_*HTA9* were amplified via PCR from tomato complementary DNAs (cDNAs) and then subcloned into *pCambia1300-35S-N1-YFP* constructs, which were confirmed by sequencing. Transient expression and image processing were conducted as previously reported^68^. The constructs containing *Sl_HTA11* and *Sl_HTA9* were introduced into *Agrobacterium tumefaciens* (GV3101) by electroporation, after which the transformed bacteria were transiently expressed in tobacco leaves. After 48 h, the tobacco leaves infiltrated with GV3101 were observed under a Delatavison Personal DV system (Applied Precision) using an Olympus UPLanApo water immersion objective lens (60 ×/1.20 numerical apertures). The filters used for DAPI included an exciter (360/40 nm/nm) and an emitter (457/50 nm/nm); for YFP, an exciter (492/18 nm/nm) and an emitter (535/30 nm/nm).

### mRNA-seq assays

Tomato fruits of WT and *sl_h2a.z* plants were harvested at the MG (34 dpa), B (40 dpa), P (43 dpa), and R (47 dpa) stages. For mRNA-seq, pericarp tissues of tomato fruits (*n* = 15) at each developmental stage from WT or *sl_h2a.z* plants were mixed together. The samples were frozen immediately in liquid nitrogen and then stored at −80 °C for further analysis. mRNA-seq was performed by Novogene, China.

Briefly, total RNA was extracted from pericarp tissue via TRIzol (Thermo Fisher) and verified on 1% agarose gels. The RNA purity was checked using a NanoPhotometer^®^ spectrophotometer (IMPLEN, USA), and the RNA integrity was assessed using an RNA Nano 6000 Assay Kit of the Bioanalyzer 2100 system (Agilent Technologies, USA). A total amount of 1 µg of RNA per sample was used as input material for the RNA sample preparations. Sequencing libraries were generated using a NEBNext^®^ Ultra^TM^ RNA Library Prep Kit for Illumina (NEB, USA), following the manufacturer’s recommendations. Three biological replicates were included for each treatment (sample). Clustering of the index-coded samples was performed on a cBot Cluster Generation System using TruSeq PE Cluster Kit v3-cBot-HS (Illumina) according to the manufacturer’s instructions. After cluster generation, the prepared libraries were sequenced on an Illumina NovaSeq platform, and 150 bp paired-end reads were generated.

### Data analysis

#### Quality control

Raw data (raw reads) in fastq format were first processed through in-house Perl scripts. Clean data (clean reads) were obtained by removing reads containing adapters, reads containing poly-N sequences and reads of low quality from the raw data. At the same time, the Q20 value, Q30 value and GC content of the clean data were calculated. All downstream analyses were based on clean data with high quality.

#### Reads mapped to the reference genome

An index of the reference genome was constructed using HISAT2 v2.0.5, and paired-end clean reads were aligned to the reference genome (Tomato genome version *SL3.0*) using HISAT2 v2.0.5^69^. We selected HISAT2 as the mapping tool because it can generate a database of splice junctions based on the gene model annotation file and thus provides mapping results that are better than those of other nonsplice mapping tools.

#### Novel transcript prediction

The mapped reads of each sample were assembled by StringTie (v1.3.3b)^70^ by a reference-based approach. StringTie uses a novel network flow algorithm as well as an optional de novo assembly step to assemble and quantitate full-length transcripts representing multiple splice variants for each gene locus.

#### Quantification of gene expression level

FeatureCounts v1.5.0-p3 was used to count the numbers of reads mapped to each gene^71^. The FPKM of each gene was then calculated based on the length of the gene and read count mapped to that gene^72^.

#### Differential expression analysis

Differential expression analysis was performed using the DESeq2 R package (1.16.1)^73^. Using a model based on the negative binomial distribution, DESeq2 provides statistical routines for determining differences in expression within digital gene expression data. The resulting *P*-values were adjusted using Benjamini and Hochberg’s approach for controlling the false discovery rate. Genes with an adjusted *P*-value < 0.05 and a | log2(fold change)|>0.0 as revealed by DESeq2 were considered differentially expressed genes.

#### GO enrichment analysis of differentially expressed genes

Gene ontology (GO) enrichment analysis of differentially expressed genes was performed using an online tool (http://geneontology.org/). GO terms with an adjusted *P*-value < 0.05 were considered significantly enriched

### Detection of carotenoids

Carotenoid contents were detected by Metware (http://www.metware.cn/) based on the AB SCIEX QTRAP 6500 liquid chromatography with tandem mass spectrometry (LC-MS/MS) platform. Four biological replicates of each treatment (group) were assessed. In brief, fresh plant materials were freeze dried and stored at −80 °C until extraction. Afterward, <50 mg of dried plant powder for each sample was extracted with a solution buffer (n-hexane:acetone:ethanol). The extract was vortexed for 20 min at room temperature, and the supernatants were collected after centrifugation. The residue was reextracted by repeating the abovementioned steps. The entire supernatant was collected and then evaporated to dryness under a nitrogen stream and reconstituted in a solution buffer (methanol:MTBE). The solution was ultimately filtered through a 0.22 μm filter for further LC-MS analysis.

### qPCR assays

Total RNA was extracted by TRIzol (Invitrogen), and a total of 1 μg of mRNA was converted to cDNA with M-MuLV reverse transcriptase (New England Biolabs). The cDNAs were used as templates for real-time PCR with iQ SYBR Green Supermix (Bio-Rad). qPCR was performed with the primers listed in Supplemental Table 7.

## Supplementary information

Dataset 1

Dataset 2

Dataset 3

Dataset 4

Dataset 5

Dataset 6

Dataset 7

Supplementary Information

## Data Availability

The mRNA-seq data generated in this publication have been deposited in the National Center for Biotechnology Information database (PRJNA663979).
