# Supplementary material for: The histone variant Sl_H2A.Z regulates carotenoid biosynthesis and gene expression during tomato fruit ripening
Source: Hortic Res. 2021 Apr 1;8:85. doi: 10.1038/s41438-021-00520-3 (PMC8012623; doi:10.1038/s41438-021-00520-3)
Supplement: Supplementary file 8 — Supplementary Information [file 41438_2021_520_MOESM8_ESM.pdf]

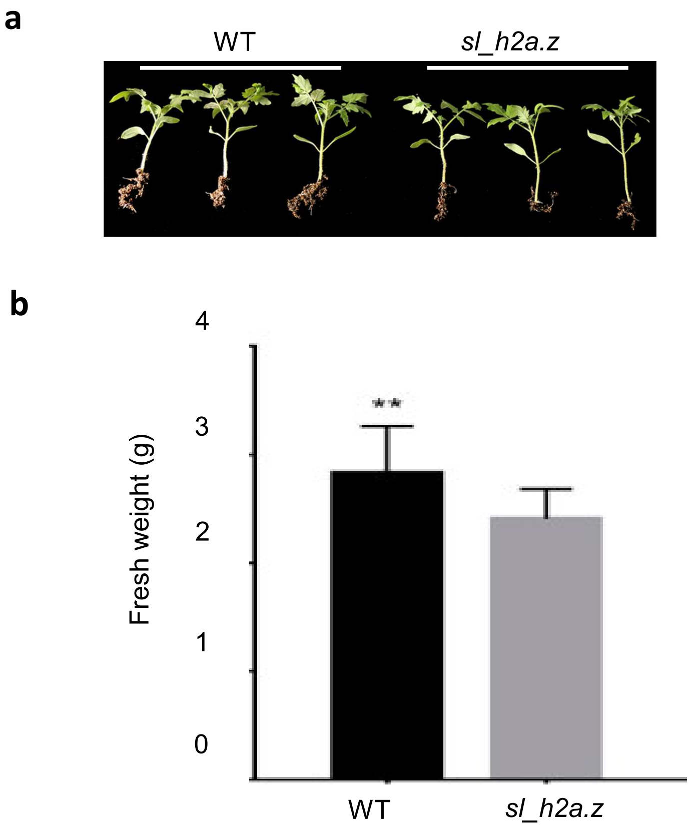

Supplemental Fig. 1 The double mutations in *Sl_H2A.Z* decrease the biomass of tomato seedlings. **a** Morphological image of 4-week-old tomato seedlings in WT and *sl_h2a.z* backgrounds. **b** The average weight of overground seedlings in WT and *sl_h2a.z* *P < 0.01 (n=3), compared with WT control (2-tailed t test).

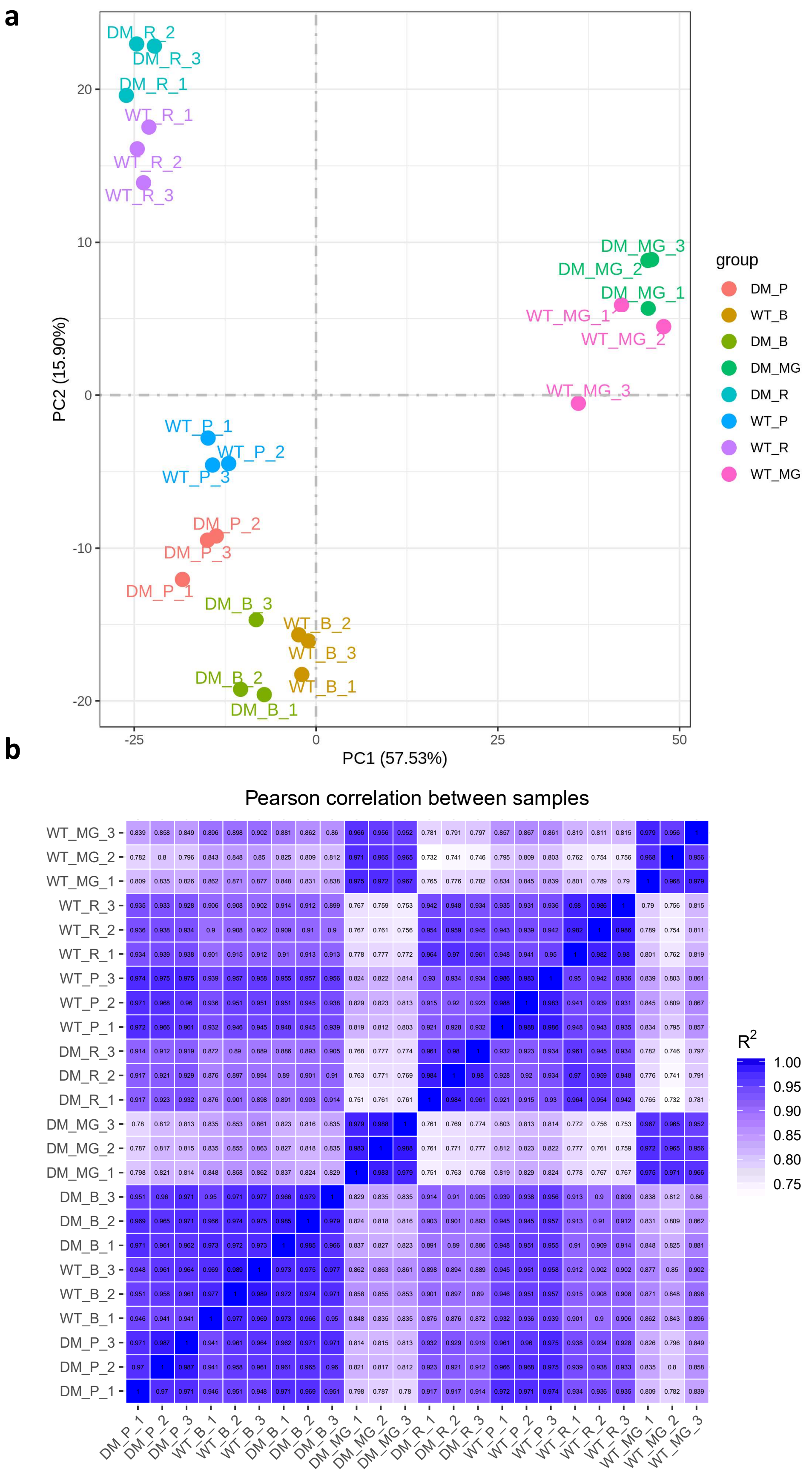

Supplemental Fig. 2 Quality control the mRNA-seq data. **a,b** PCA results (**a**) and Pearson correlation index (**b**) showing consistency among the three biological replicates of mRNA-seq samples. DM indicates *sl_h2a.z* double-mutant.

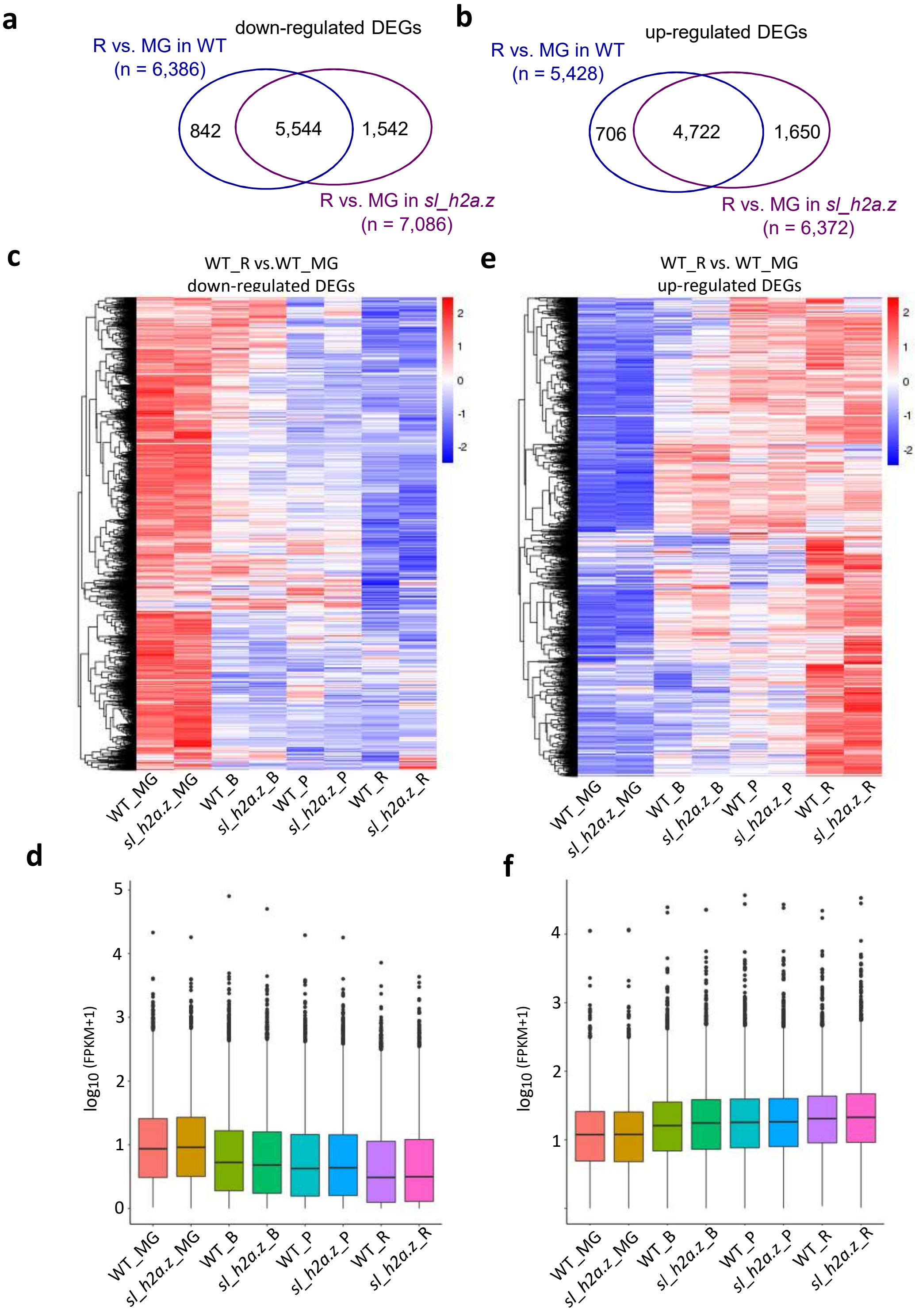

Supplemental Fig. 3 Fruit ripening and dysfunction of *Sl*_H2A.Z reprogram the pattern of gene expression. **a, b** Overlap of down-regulated DEGs (**a**) and up-regulated DEGs (**b**) in the comparisons of WT_R vs. WT_MG and *sl_h2a.z*_R vs. *sl_h2a.z*_MG. **c, d** Heatmap representation (**c**) and boxplot representation (**d**) of the overlapped down-regulated DEGs by WT_R vs. WT_MG and *sl_h2a.z*_R vs. *sl_h2a.z*_MG. **e, f** Heatmap representation (**e**) and boxplot representation (**f**) of the overlapped up-regulated DEGs by WT_R vs. WT_MG and *sl_h2a.z*_R vs. *sl_h2a.z*_MG.

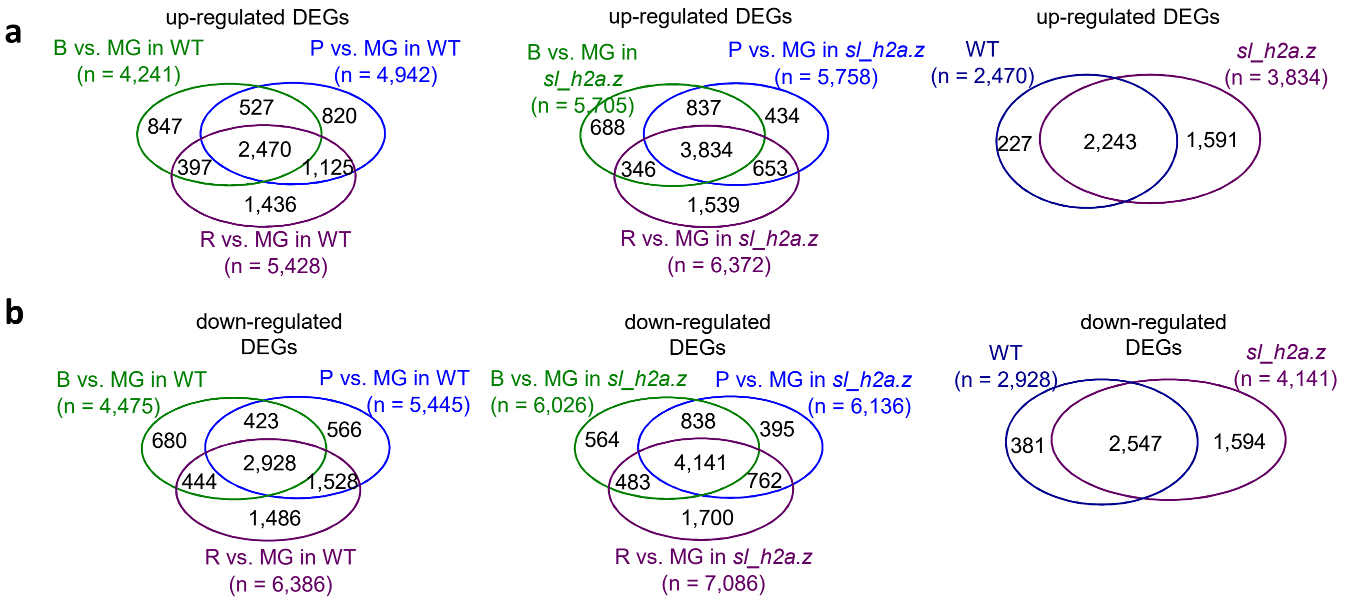

Supplemental Fig. 4 Ripening- and H2A.Z-dependent DEGs in WT and *sl_h2a.z* tomato fruits. **a** The overlap between the ripening-dependent up-regulated DEGs in WT fruits and *sl_h2a.z* double-mutant fruits. The up-regulated DEGs commonly shared by the comparisons of B vs. MG, P vs. MG, and R vs. MG are referred to as the ripening-dependent up-regulated DEGs in WT fruits (left panel) and in *sl_h2a.z* fruits (middle panel). **b** The overlap between the ripening-dependent down-regulated DEGs in WT fruits and *sl_h2a.z* double-mutant fruits. The down-regulated DEGs commonly shared by the comparisons of B vs. MG, P vs. MG, and R vs. MG are referred to as the ripening-dependent down-regulated DEGs in WT fruits (left panel) and in *sl_h2a.z* fruits (middle panel).

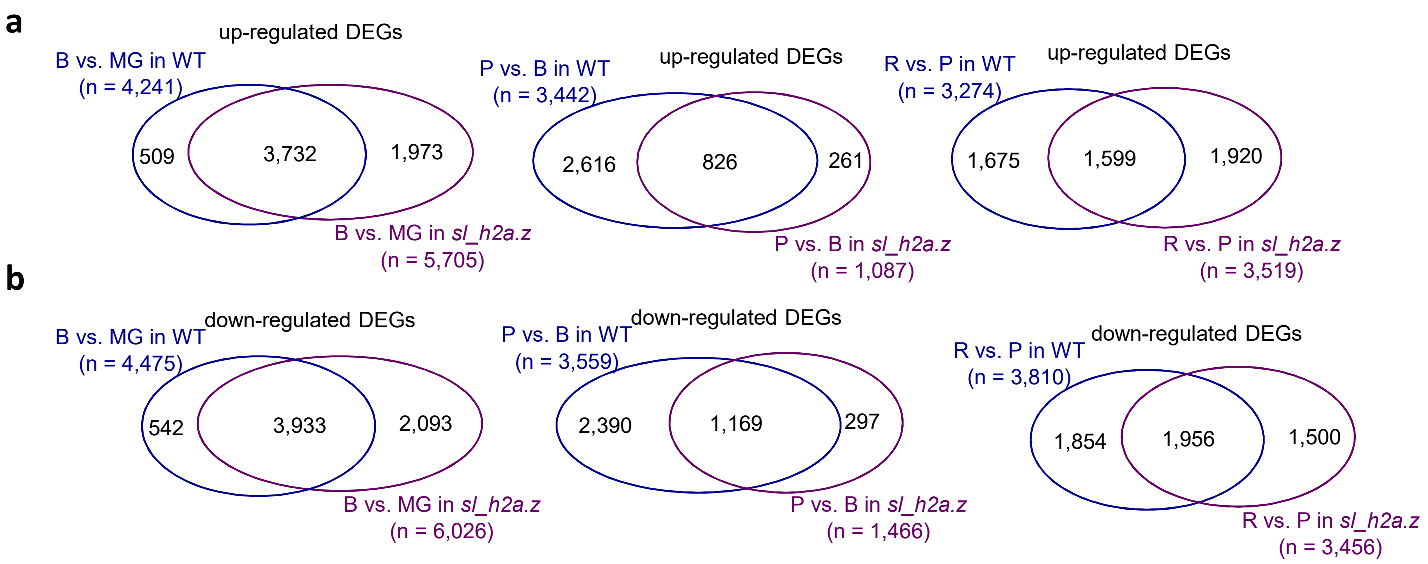

Supplemental Fig. 5 Characterization of DEGs identified in the WT and *sl_h2a.z* fruits during fruit ripening. **a, b** Overlap of up-regulated DEGs (**a**) and down-regulated DEGs (**b**) between the indicated comparisons in WT and *sl_h2a.z* fruits. Samples at the two nearby stages during ripening were analyzed, including B vs. MG stage (left panel), P vs. B stage (middle panel), and R vs. P stage (right panel).

a

Components	Equation	R^2
(E/Z)-Phytoene	y = 0.02323 x + 7.20801e-4	0.998
Lycopene	y = 0.16141 x + 8.27726e-4	0.999
α-Carotene	y = 0.35893 x + 0.00122	0.998
β-Carotene	y = 0.62752 x + 0.00562	0.998
γ-Carotene	y = 0.51502 x + -0.00274	0.998
ε-Carotene	y = 1.46532 x + 0.00013	0.999
Lutein	y = 0.93282 x + 0.00463	0.999
β-Cryptoxanthin	y = 0.34449 x + 0.00821	0.997
α-Cryptoxanthin	y = 1.00961 x + 0.00299	0.999
Apocarotenal	y = 9.04559 x + -1.24542	0.999
Neoxanthin	y = 2.48211 x + -0.00830	0.998
Zeaxanthin	y = 0.19627 x + 0.00647	0.997
Violaxanthin	y = 3.18262 x + 0.00535	0.995

c

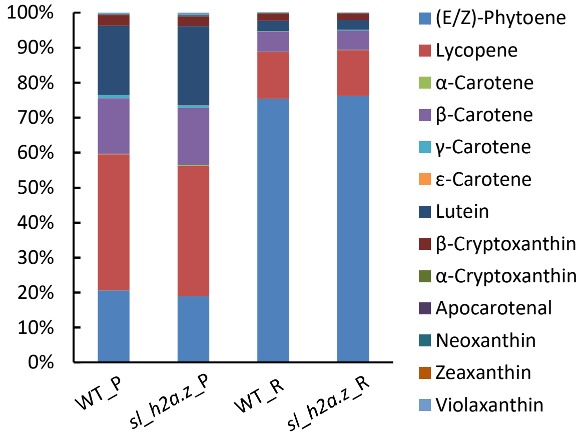

b

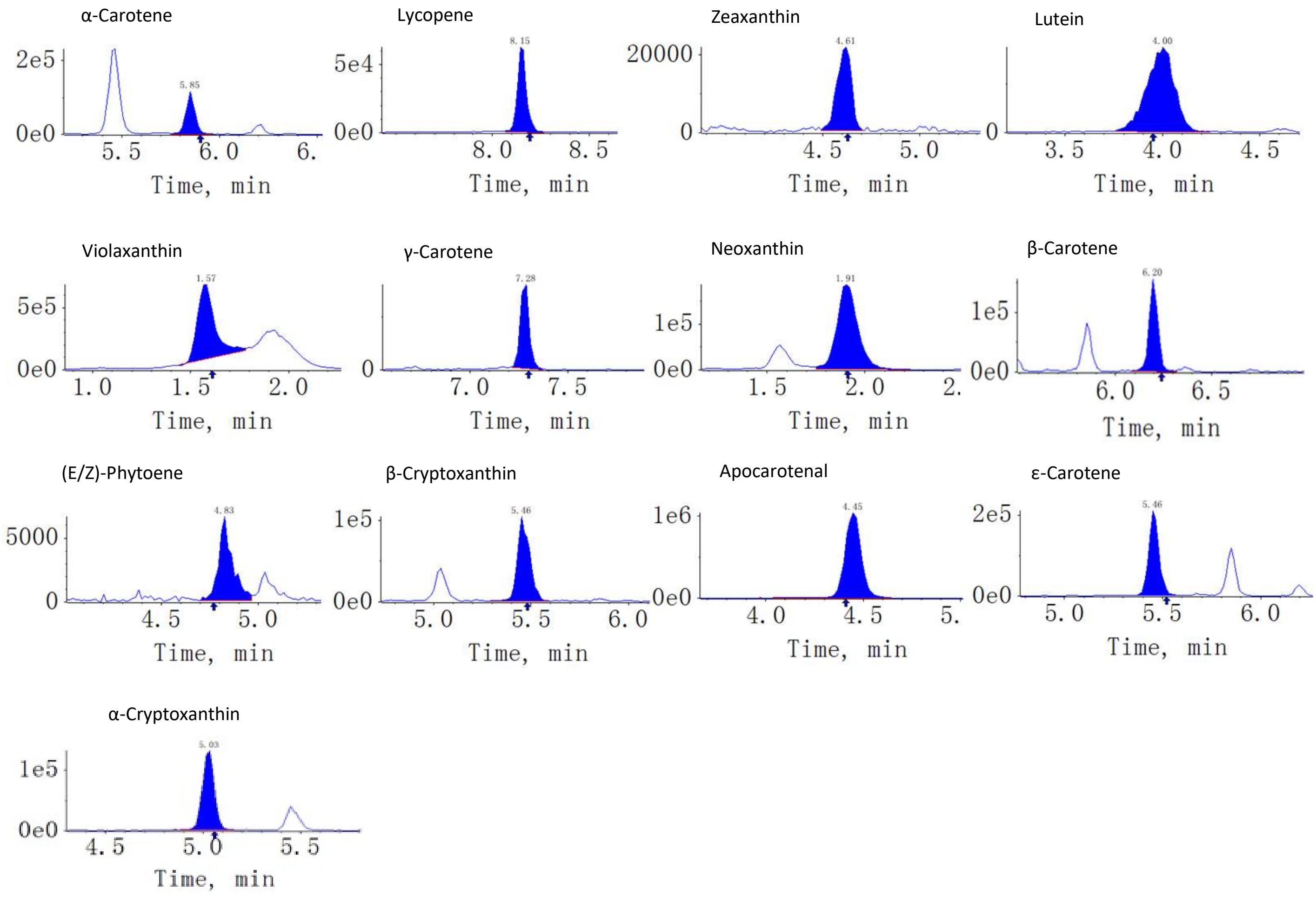

Supplemental Fig. 6 Dysfunction of *Sl*_H2A.Z regulates the contents of carotenoids. **a** The equation and R^2 of the indicated standard components of carotenoids. **b** The curve and peak pattern of the indicated standard components of carotenoids. **c** The percentage of the indicated components in the WT and *sl_h2a.z* fruits at P and R stage.
